# Supplementary material for: Quantification of ligand and mutation-induced bias in EGFR phosphorylation in direct response to ligand binding
Source: Nat Commun. 2023 Nov 21;14:7579. doi: 10.1038/s41467-023-42926-8 (PMC10663608; doi:10.1038/s41467-023-42926-8)
Supplement: Supplementary file 1 — Supplementary Information [file 41467_2023_42926_MOESM1_ESM.pdf]

SUPPLEMENTARY INFORMATION

Quantification of ligand and mutation-induced bias in EGFR phosphorylation in direct response to ligand binding

Daniel Wirth, Ece Özdemir and Kalina Hristova

Department of Materials Science and Engineering and Institute for NanoBioTechnology, Johns Hopkins University, 3400 Charles Street, Baltimore, MD 21218;

Supplementary Figures

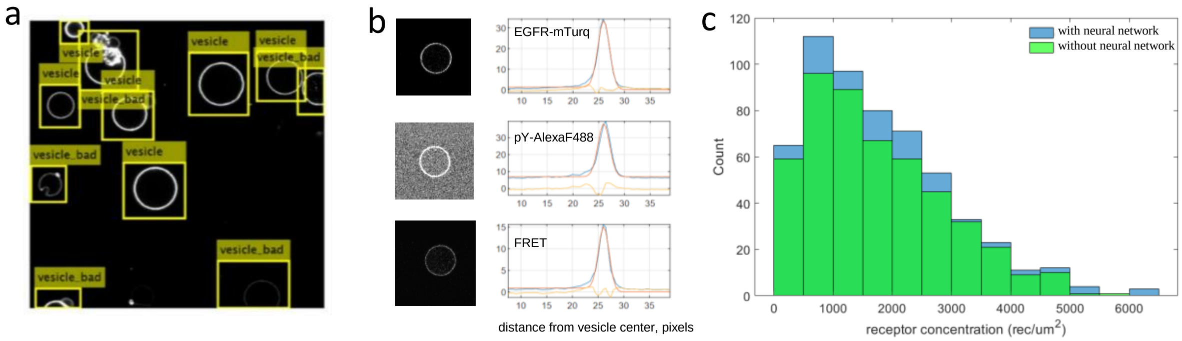

Supplementary Figure 1. Neural network recognition and analysis of plasma-membrane derived vesicles with FGFR3-eYFP. a) Typical result of the neural network analysis. The neural network is able to recognize good and bad vesicles of different intensities. b) Fluorescence intensity calculations for a single vesicle. Intensities per unit membrane area were obtained by integrating the Gaussian intensity profiles across the membrane, shown as a function of the distance from the vesicle center. Blue: experimentally measured profile. Red: fit to the experimentally measured profile, composed of a Gaussian intensity which is integrated and analyzed, and background. Yellow: residual between experimental profiles and fits. c) Comparison of histograms of FGFR3-eYFP concentrations from vesicles that are pre-selected by the neural network (blue) and the ones pre-selected manually by a trained researcher (green). The two analysis methods show good agreement.

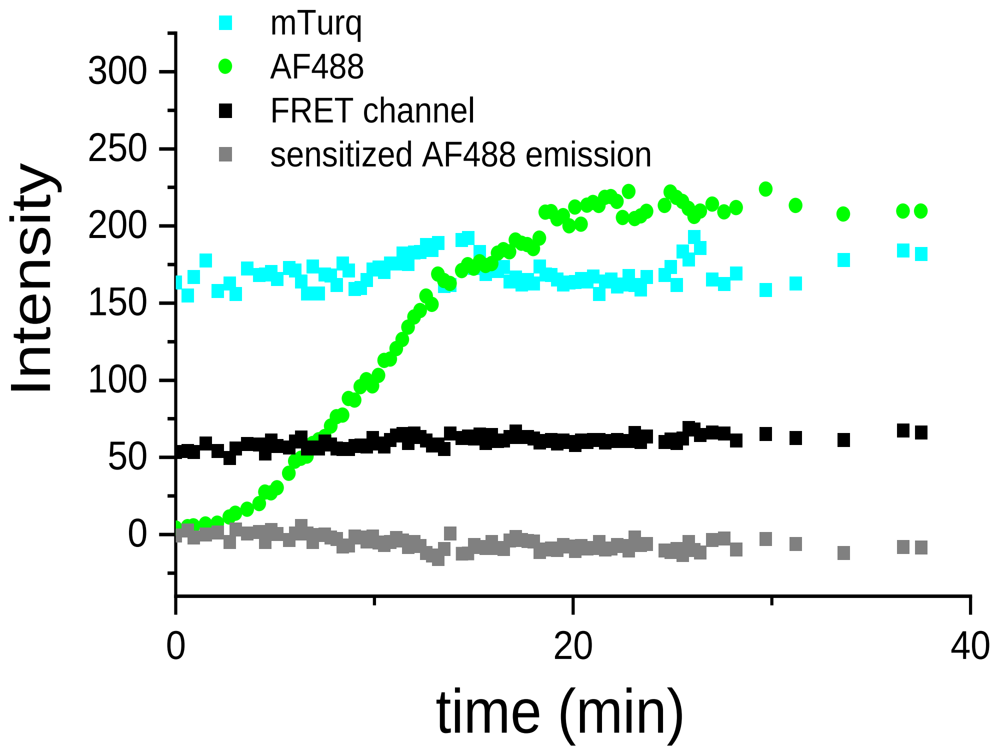

Supplementary Figure 2. Kinetics traces acquired for EGFR in response to TGFα. FRET is measured concomitantly with measurements of EGFR and antibody concentrations on the membrane. The sensitized acceptor emission (grey) is calculated from the measured intensity in the FRET channel (black) after correcting for donor bleed-though and for direct acceptor emission. There is no sensitized acceptor emission and there is no decrease in donor fluorescence, indicative of zero FRET. Exemplary kinetic traces for one vesicle are shown.

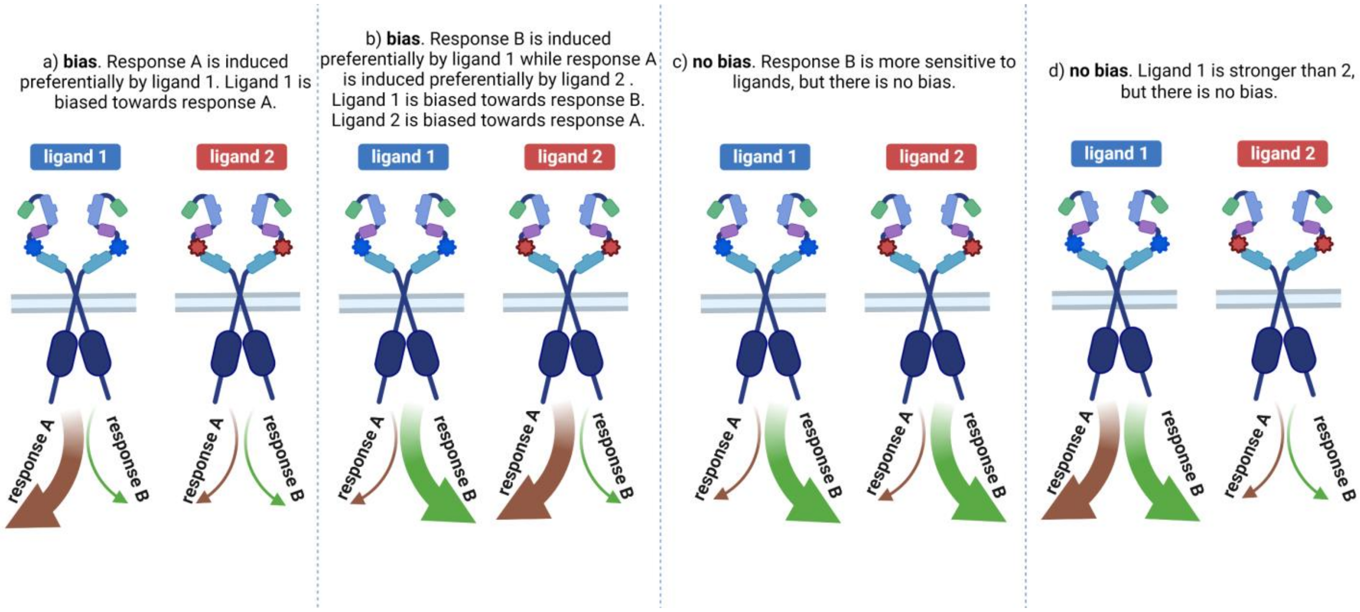

Supplementary Figure 3. A schematic diagram showing two cases of bias (a and b) and two cases of no bias (c and d).

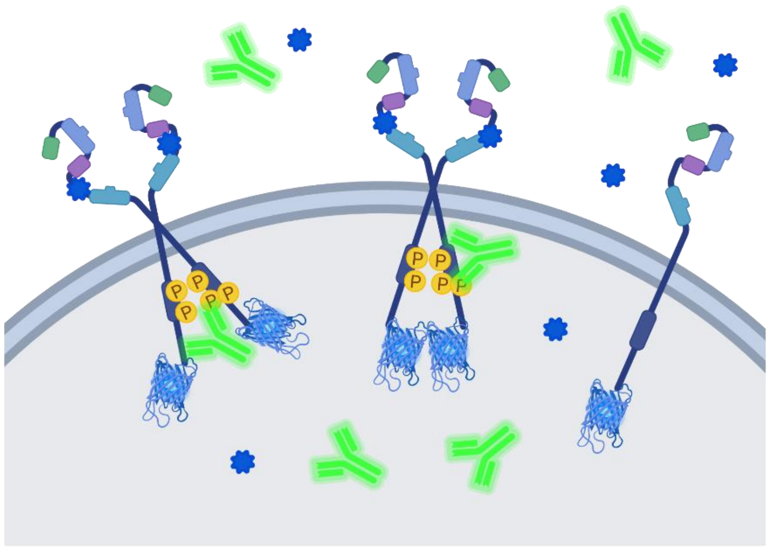

Supplementary Figure 4. The experimental set up to follow receptor phosphorylation in plasma membrane derived vesicles which are permeant to macromolecules. RTKs in the vesicle membrane dimerize and get phosphorylated in response to ligand (blue star) binding. In the presence of ligand and ATP cocktail, a fluorescent specific anti-phosphoY antibody (green) is recruited to the membrane, and its recruitment is quantified, along with RTK concentrations in each vesicle.

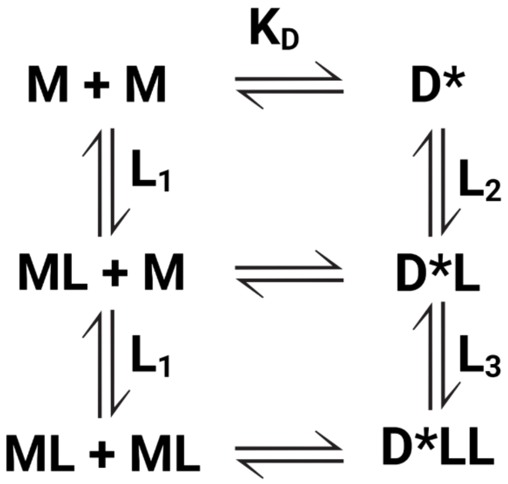

Supplementary Figure 5. The thermodynamic cycle describing EGFR activation. * indicates that the dimeric receptors can be phosphorylated.

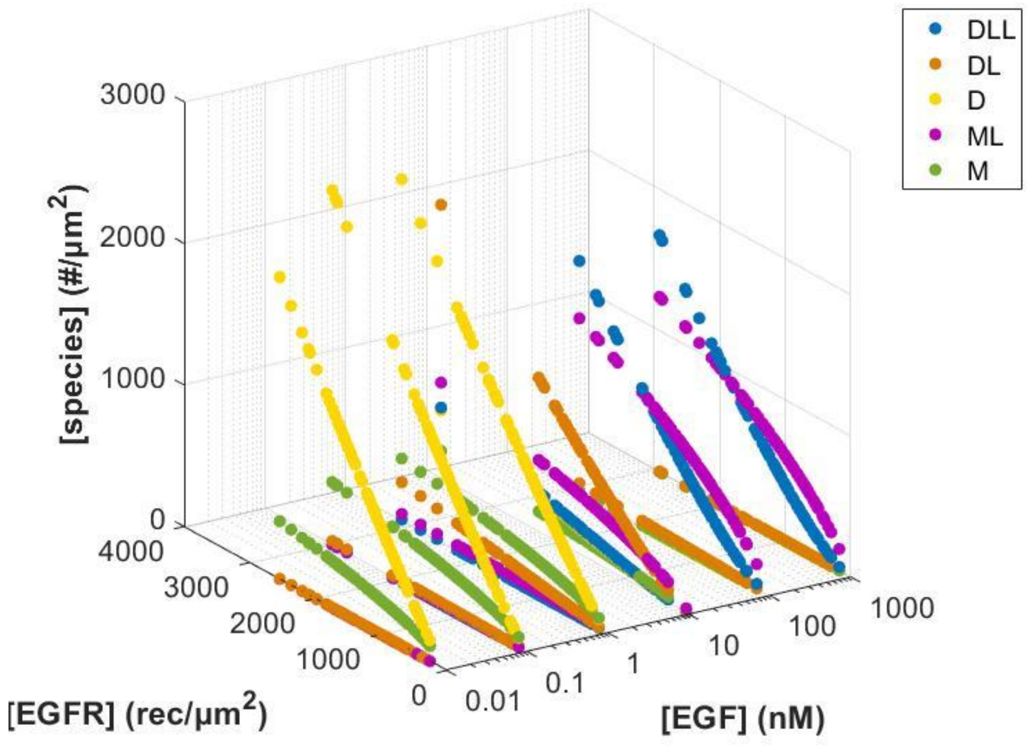

Supplementary Figure 6. Concentrations of WT EGFR monomers and dimers, as a function of receptor and EGF concentrations. The monomer (green), unliganded dimer (yellow), liganded monomer (magenta), single-liganded dimer (orange), and double-liganded dimer (blue) distributions are plotted as a function of EGFR concentration (y-axis) and EGF concentration (x-axis) for every vesicle. The unliganded dimer concentrations (yellow) are used to correct the measured response in Figures 2A and 3A. Data shown in this figure is derived from 986 vesicles in 3 independent experiments.

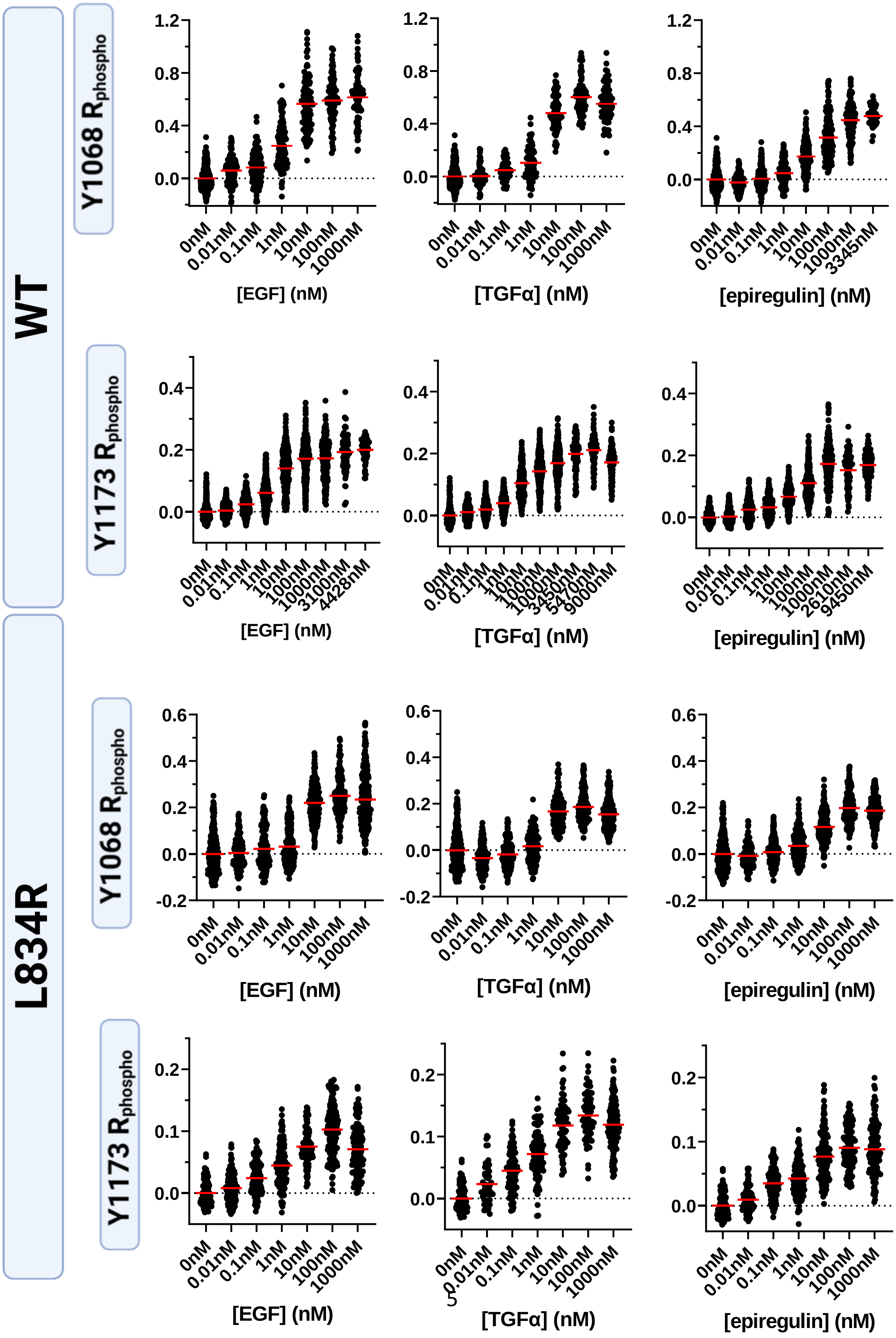
WT
L834R
Y1068 $R_{phospho}$
Y1173 $R_{phospho}$
[EGF] (nM)
[TGFα] (nM)
[epiregulin] (nM)
0nM
0.01nM
0.1nM
1nM
10nM
100nM
1000nM
3345nM
3100nM
4428nM
3450nM
5470nM
9000nM
2610nM
9450nM
1.2
0.8
0.4
0.0
0.2
0.6
-0.2
0.1

Supplementary Figure 7. WT and L834R EGFR phosphorylation in individual vesicles in response to EGF, TGFα, and epiregulin, after correction for basal phosphorylation. The red center bar represents the mean. Data shown in this figure were derived from 19579 vesicles in 48 independent experiments.

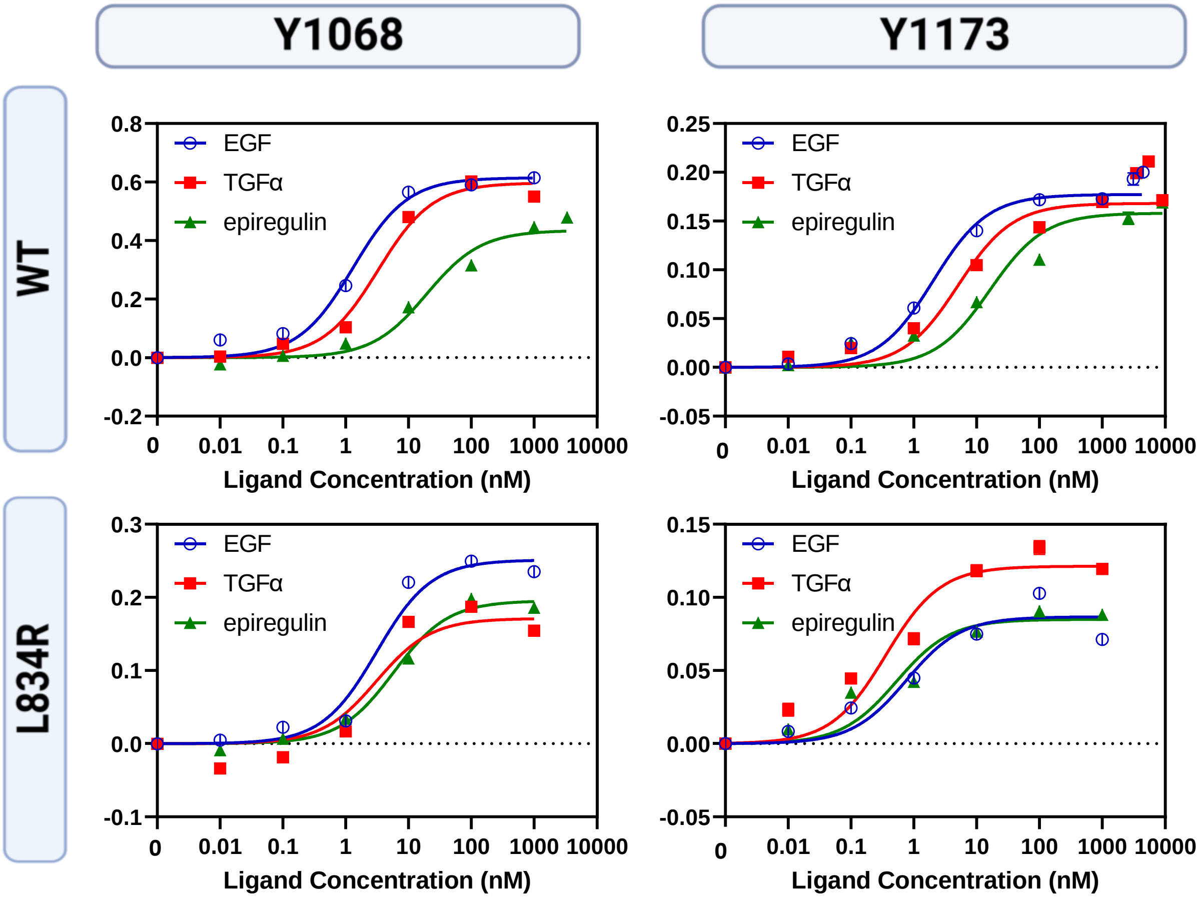

Supplementary Figure 8. Fitted corrected dose-response curves for WT and L834R EGFR, for the three ligands. Shown are mean and standard errors (if errors are not visible, they are smaller than the symbols). The solid lines are fits to all the single vesicle data in Figures 2A and 3A (19579 vesicles in 48 independent experiments) using equation (1).

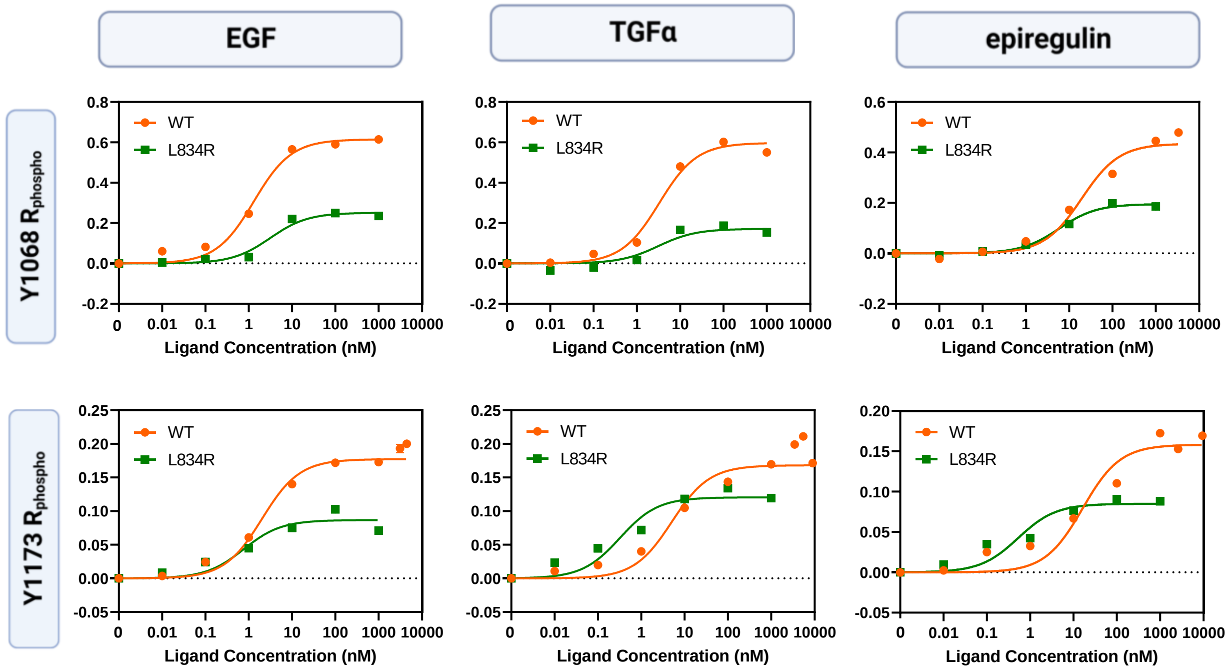

<u>Supplementary Figure 9. Comparison of corrected fitted dose response curves for WT and L834R EGFR in response to EGF, TGFα, and epiregulin.</u> Same curves as in Supplementary Figure 8, replotted to show the difference in phosphorylation between the wild-type and the mutant. Shown are means and standard errors (if errors are not visible, they are smaller than the symbols). The solid lines are fits to all the single vesicle data in Figures 2A and 3A (19579 vesicles in 48 independent experiments) using equation (1).

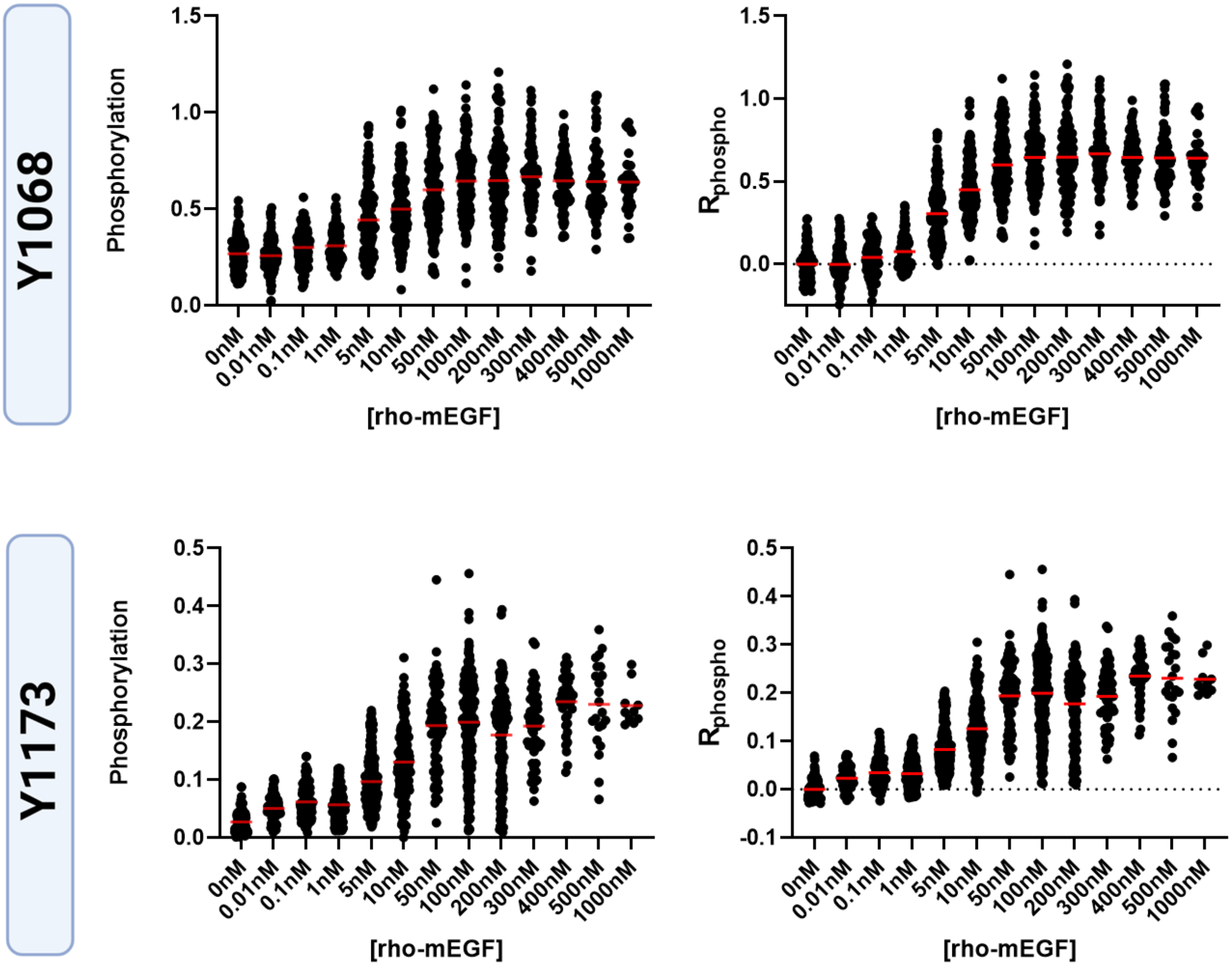

Supplementary Figure 10. Measured and corrected EGFR dose responses to rho-mEGF. Individual vesicle datapoints are shown (n=2173 for Y1068 and n=1987 for Y1173, 9 independent experiments). The red center bar represents the mean.

Supplementary Tables

Supplementary Table 1. EC_{50} and E_{top} for the EGF, TGFa and epireglin responses. Best-fit values ± SEM are shown.

		ligand	EC_{50} (nM)	E_{top}
**WT**	**Y1068**	EGF	1.4 ± 0.1	0.61 ± 0.01
		TGFα	3.4 ± 0.3	0.59 ± 0.01
		epiregulin	19.5 ± 2.0	0.43 ± 0.01
	**Y1173**	EGF	2.3 ± 0.1	0.19 ± 0.01
		TGFα	5.2 ± 0.3	0.17 ± 0.01
		epiregulin	16.2 ± 1.2	0.16 ± 0.01
**L858R**	**Y1068**	EGF	3.1 ± 0.3	0.25 ± 0.01
		TGFα	3.2 ± 0.4	0.17 ± 0.01
		epiregulin	5.8 ± 0.6	0.20 ± 0.01
	**Y1173**	EGF	0.7 ± 0.1	0.08 ± 0.01
		TGFα	0.3 ± 0.1	0.12 ± 0.01
		epiregulin	0.7 ± 0.1	0.09 ± 0.01

Supplementary Table 2. EC_{50} and E_{top} for the rho-mEGF response. Best-fit values ± SEM are shown.

	EC_{50} (nM)	E_{top}
**Y1068**	5.5 ± 0.3	0.67 ± 0.01
**Y1173**	6.8 ± 0.4	0.21 ± 0.01

Supplementary Table 3. Calculated Rho-mEGF bias coefficients. Ordinary one-way ANOVA was used for p-value calculation. Tukey test was used to account for multiple comparisons. Reported are p values adjusted for multiple comparisons.

lig vs rho-mEGF	$ß_{lig}$ (Y1068 vs Y1173)	p-value (adjusted)
**EGF**	0.12 ± 0.05	0.48
**TGFα**	0.13 ± 0.06	0.29
**epiregulin**	-0.23 ± 0.06	0.0001

Supplementary Notes

Ligand bias

The term "ligand bias" describes the ability of ligands to preferentially activate a subset of signaling pathways, which can lead to fundamentally different biological responses (6, 7). Ligand bias does not simply reflect differences in potency (EC_{50}, the concentration of ligand that induces 50% of the maximum response), or efficacy (E_{top}, the maximum possible response due to a ligand). These differences represent quantitative differences in signaling, while bias represents fundamental (often called "qualitative") differences in signaling (8-10). This is illustrated in Supplementary Figure 3 for two different ligands (blue and red) and two distinct responses (A, brown and B, green). Panels a and b show two cases of ligand bias while panels b and c show two cases of no bias. In Supplementary Figure 3a both ligands activate response B similarly, but ligand 1 induces response A more efficiently than ligand 2. In Supplementary Figure 3b ligand 1 preferentially induces response B while ligand 2 exhibits a preference towards response A. Supplementary Figure 3b and c show only quantitative differences and therefore the ligands are not biased. Supplementary Figure 3b depicts the case where response B is more efficiently triggered than response A, but the two ligands act similarly without bias. In Supplementary Figure 3c ligand 1 activates both responses more efficiently than ligand 2, however the difference of activation in the two responses is the same for the two ligands and thus there is no bias. A variation of the last case would be where the efficiency of response B vary proportionally to the efficiency of response A. In such a case there would be no ligand bias, either.

The concept of ligand bias complements and expands concepts in traditional pharmacology where ligands are classified as either an agonist, antagonist or inverse agonist (11). A biased ligand can be either an agonist, antagonist or inverse agonist (12).

There have been numerous reports that propose the existence of ligand bias for FGF (13, 14), TRK (15-20), ERBB (21), Eph (22, 23), and PDGF (24) receptors. However, bias coefficients have not been calculated in these studies and the degree of the bias is unknown. Furthermore, it has been

shown that different concentrations of adaptor proteins can bias signaling (25). This so-called "system bias" is often difficult to account for and decouple in cellular studies (26).

Supplementary Methods

Identification of vesicles using machine learning, and quantification of their fluorescence intensities

In this work, we had to analyze confocal images of thousands of vesicles. To increase the through-put of data processing, we developed an approach that uses artificial intelligence. The trained neural network is able to separate "vesicles" from "bad vesicles" and "cell debris". A "vesicle" is defined as a spherical vesicle, completely separated from its parent cell (Supplementary Figure 1a). Vesicles that are out of focus, cut off by image edges, non-circular, or attached to cells were classified as "bad vesicles". Remnants of the cells after vesiculation were classified as "cell debris".

For the neural network training, we used the imageLabeler app in Matlab to annotate rectangular regions in confocal micrographs for the classes (i) vesicle, (ii) bad vesicle, and (iii) cell debris. The resulting dataset of 1402 labeled objects was randomly shuffled and split into 70% training data and 30% test data. To build a neural network that identifies good vesicles, the FasterRCNN object detection network based on ResNet-18 was trained with the training data in Matlab. ResNet-18's res4b_relu layer was chosen as the feature extraction layer. The learning rate was set to 0.001 and images were augmented during the training process by random horizontal reflection. 15 predefined anchor boxes, calculated using Matlab's "estimateAnchorBoxes", were used. Bounding box overlap ratios were set to 0.6 – 1 for positive training samples and to 0 – 0.3 for negative training samples to ensure a tight overlap with ground truth. The network was trained for a total of 10 epochs.

To train the network, we analyzed images of ~1400 vesicles with FGFR3-eYFP, an RTK that has been used in prior work for method development (1, 2). Supplementary Figure 1a shows the result of running the neural network on an image of FGFR3-eYFP vesicles. The neural network was able to distinguish between good and bad vesicles over broad intensity ranges.

Once selected by the neural network, the vesicle images were processed with a Matlab program (3, 4), which finds the center of the vesicle and performs a check to ensure that only spherical, defect-free vesicles are included in the analysis. Once the center is found, the intensity profile as a function from the center is plotted and the membrane intensity is calculated (Supplementary Figure 1b). To accomplish this, the signal that originates from the membrane is fitted to a Gaussian function, and the background fluorescence to an error function (Supplementary Figure 1b).

We found that the vesicle analysis program could not be efficiently applied for images acquired with an automated stage, without the pre- selection performed by the neural network. The vesicle analysis program recognized vesicles based on a threshold that is referenced to the object of highest intensity (5). Many images from the automated imaging sessions contained multiple vesicles of different intensities, and the brighter vesicles precluded the identification of vesicle of weaker intensities.

After the neural network recognition, each sub-image of a good vesicle, such as the ones shown in Supplementary Figure 1a, was inputted into the vesicle analysis program. In this case, there was

only a single vesicle per sub-image. Thus, each vesicle selected by the neural network was evaluated separately with a background threshold determined by its own intensity. As a result of this evaluation, each vesicle from the neural network was either rejected or analyzed further as shown in Supplementary Figure 1b.

To validate the neural network, we manually selected and cropped images from an automated imaging session and we ran the vesicle analysis program. In parallel, we used the neural network on the same data set to pre-select the images. After analysis, the receptor concentration was calculated for each vesicle (3) and the results are shown in Supplementary Figure 1c. The histograms of data in Supplementary Figure 1c show that the neural network performs similarly to a trained researcher who manually selects and crops images of good vesicles.

In the case of automatic imaging, many images did not include any good vesicles. These were rejected by the neural network and were never inputted into the vesicle analysis program, significantly speeding up the analysis. Thus, the addition of the neural network made the automatic imaging and analysis highly efficient.

The neural network approach was used as a first step in the quantification of both EGFR phosphorylation in Figures 2A and 3A and of ligand binding in Figure 5. It was also used to quantify the degree of penetration by dextrans into the vesicles in Figure 1B. The dextran experiments demonstrated the presence of defects of vesicles, which ensured that the phosphorylated tyrosines are accessible to externally added antibodies.

Correction of dose response curves for basal EGFR phosphorylation

According to the canonical model of RTK activation, RTKs are monomeric in the absence of ligand and are crosslinked upon ligand binding, which brings their catalytic domains in close proximity (27). However, numerous studies have demonstrated that EGFR activation is much more complex. First, it has been reported that EGFR can form dimers even in the absence of ligand, and that these dimers are phosphorylated, although they are not able to initiate downstream signaling cascades (28). Second, there are reports that EGFR can form oligomers upon ligand binding (29, 30). However, other studies show that EGFR exists predominantly in dimeric form (31).

To collect dose-response curves, phosphorylation of EGFR on the vesicle membrane was measured, as illustrated in Supplementary Figure 4, over a broad range of ligand concentrations. These dose response curves, shown in Figures 2A and 3A in the main text, capture the phosphorylation of unliganded dimers, single-liganded dimers, and double-liganded EGFR dimers. The unliganded dimers account for basal phosphorylation of EGFR at zero ligand. Since we are interested in the response of EGFR to ligand, we modeled the concentration of unliganded EGFR dimers, and we corrected the dose response curves for the basal phosphorylation. This was done in order to calculate bias coefficients and therefore provide quantitative support for the bias plots, which rely on visual inspection (26).

Despite open questions about the association state of EGFR, it has been shown that its function can be described in quantitative terms by the thermodynamic cycle in Supplementary Figure 5 which accounts for the coupling between ligand binding and EGFR dimerization (32). This cycle shows how increases in receptor and ligand concentration drive the formation of double-liganded EGFR dimers, and is used here to determine the unliganded dimer concentrations in each vesicle.

In our experiments, we know EGFR concentration in the membrane of each vesicle, $[R_t]$. We also know the total EGFR concentration in the imaging dish, $[R_{tot}]$, and the total ligand concentration in the imaging dish $[L_{tot}]$. The dimerization constants K_D for EGFR and the L834R mutant have been measured as $K_D = 6.4x10^{-3}$ μm^2 and $3.7x10^{-2}$ μm^2, respectively, in the plasma membrane-derived vesicles (28). Noteworthy, the dimerization constant has also been measured in cells, and is the same (32). The EGF binding constants for the monomer, L_1, the unliganded dimer, $L_{2,}$ and the liganded dimer, $L_{3,}$ have been measured as $L_1 = 4.6x10^9$ M^{-1}, $L_2 = 5.3x10^9$ M^{-1}, and $L_3 = 3.4x10^8$ M^{-1} (32). While both EGF and TGFα are known as high-affinity ligands for EGFR, with similar association constants, epiregulin is known as a low-affinity ligand, and its association constant has been reported to be 46 times lower than that of EGFR (21, 33). The mouse EGF-human EGFR association constant is 3 times lower than the human EGF association constant (34).

Based on the cycle in Supplementary Figure 5, the concentrations of unliganded monomers, M, unliganded dimers, D, liganded monomers, ML, single-liganded dimers, DL, and double-liganded dimers, DLL, can be written as a function of these known equilibrium constants, the monomer concentration [M], and the free ligand concentration $[L_{free}]$ according to:

$$[D] = K_D * [M]^2 \quad \text{(Supplementary Equation 1)}$$

$$[ML] = L_1 * [L_{free}] * [M] \quad \text{(Supplementary Equation 2)}$$

$$[DL] = L_2 * [L_{free}] * K_D * [M]^2 \quad \text{(Supplementary Equation 3)}$$

$$[DLL] = L_2 * L_3 * [L_{free}]^2 * K_D * [M]^2 \quad \text{(Supplementary Equation 4)}$$

The total receptor concentration $[R_t]$ can be written as:

$$\begin{aligned}[R_t] &= [M] + [ML] + 2[D] + 2[DL] + 2[DLL] \\ &= [M] + L_1 * [L_{free}] * [M] + 2K_D * [M]^2 + 2L_2 \\ &* [L_{free}] * K_D * [M]^2 + 2L_2 * L_3 * [L_{free}]^2 * K_D \\ &* [M]^2\end{aligned} \quad \text{(Supplementary Equation 5)}$$

which can be rewritten as:

$$\begin{aligned}[R_t] &= (1 + L_1 * [L_{free}]) * [M] \\ &+ (2K_D + 2L_2 * K_D * [L_{free}] + 2L_2 * L_3 * K_D \\ &* [L_{free}]^2) * [M]^2\end{aligned} \quad \text{(Supplementary Equation 6)}$$

Thus, to be able to determine all the unknowns, we need to determine the free ligand concentration in the dish [L_{free}] and the EGFR monomer concentration [M] in each vesicle.

First, we solve for the free ligand concentration in the dish. The total number of ligand molecules in the dish L_{tot} is given by:

$$\mathrm{L_{tot}} = [\mathrm{L_{free}}] * \mathrm{volume} + \mathrm{RL_{tot}} \qquad \text{(Supplementary Equation 7)}$$

where RL_{tot} the number of ligand molecules bound to receptors in the dish. 'volume' is the volume of the vesicle solution in the dish.

RL_{tot} is the sum of ligand molecules bound to monomers ML_{tot}, single-liganded dimers DL_{tot}, and double-liganded dimers DLL_{tot}:

$$\mathrm{RL_{tot}} = \mathrm{ML_{tot}} + \mathrm{DL_{tot}} + 2\mathrm{DLL_{tot}} \qquad \text{(Supplementary Equation 8)}$$

which can be written in terms of concentrations according to:

$$\mathrm{RL_{tot}} = \mathrm{R_{tot}} * \left(\frac{[\mathrm{ML_{tot}}]}{[\mathrm{R_{tot}}]} + \frac{[\mathrm{DL_{tot}}]}{[\mathrm{R_{tot}}]} + \frac{2[\mathrm{DLL_{tot}}]}{[\mathrm{R_{tot}}]}\right) \qquad \text{(Supplementary Equation 9)}$$

Next, we assume that the average concentrations in the imaging dish is the same as the concentrations in a vesicle with an average total receptor concentration [R_{avg}]. We can write:

$$[\mathrm{R_{avg}}] = \frac{\sum_{i=1}^{n}[\mathrm{M}]_i + 2[\mathrm{D}]_i + [\mathrm{ML}]_i + 2[\mathrm{DL}]_i + 2[\mathrm{DLL}]_i}{n} \qquad \text{(Supplementary Equation 10)}$$

$$[\mathrm{ML_{avg}}] = \frac{\sum_{i=1}^{n}[\mathrm{ML}]_i}{n} \qquad \text{(Supplementary Equation 11)}$$

$$[\mathrm{DL_{avg}}] = \frac{\sum_{i=1}^{n}[\mathrm{DL}]_i}{n} \qquad \text{(Supplementary Equation 12)}$$

$$[\mathrm{DLL_{avg}}] = \frac{\sum_{i=1}^{n}[\mathrm{DLL}]_i}{n} \qquad \text{(Supplementary Equation 13)}$$

where $[x]_i$ is the concentration in the ith vesicle, and n the total number of vesicles. We can substitute (Supplementary Equation 11), (Supplementary Equation 12) and (Supplementary Equation 13) into (Supplementary Equation 9) and write:

$$\mathrm{RL_{tot}} = \mathrm{R_{tot}} * \left(\frac{[\mathrm{ML_{avg}}]}{[\mathrm{R_{avg}}]} + \frac{[\mathrm{DL_{avg}}]}{[\mathrm{R_{avg}}]} + \frac{2[\mathrm{DLL_{avg}}]}{[\mathrm{R_{avg}}]}\right) \quad \text{(Supplementary Equation 14)}$$

Note that in (Supplementary Equation 14) the units of concentration are receptors per unit area instead of receptors per volume, as the receptors are confined in the 2-dimensional plasma membrane of the vesicle. Substituting (Supplementary Equation 1), (Supplementary Equation 2), (Supplementary Equation 3), and (Supplementary Equation 4) into (Supplementary Equation 14), we obtain:

$$\mathrm{RL_{tot}} = \mathrm{R_{tot}} * \left(\frac{\mathrm{L_1} * [\mathrm{L_{free}}] * [\mathrm{M_{avg}}]}{[\mathrm{R_{avg}}]} + \frac{2\mathrm{L_2} * [\mathrm{L_{free}}] * \mathrm{K_D} * [\mathrm{M_{avg}}]^2}{[\mathrm{R_{avg}}]} + \frac{4\mathrm{L_2} * \mathrm{L_3} * [\mathrm{L_{free}}]^2 * \mathrm{K_D} * [\mathrm{M_{avg}}]^2}{[\mathrm{R_{avg}}]}\right) \quad \text{(Supplementary Equation 15)}$$

Substitution of (Supplementary Equation 15) into (Supplementary Equation 7) and further simplification yields:

$$\mathrm{L_{tot}} = \mathrm{R_{tot}} * \left(\frac{4\mathrm{L_2} * \mathrm{L_3} * \mathrm{K_D} * [\mathrm{M_{avg}}]^2}{[\mathrm{R_{avg}}]}\right) * [\mathrm{L_{free}}]^2 + \left(\mathrm{R_{tot}} * \left(\frac{2\mathrm{L_2} * \mathrm{K_D} * [\mathrm{M_{avg}}]^2}{[\mathrm{R_{avg}}]} + \frac{\mathrm{L_1} * [\mathrm{M_{avg}}]}{[\mathrm{R_{avg}}]}\right) + \mathrm{volume}\right) * [\mathrm{L_{free}}] \quad \text{(Supplementary Equation 16)}$$

(Supplementary Equation 16) and (Supplementary Equation 6) are two equations for the two unknowns, $[L_{free}]$ and $[M_{avg}]$. We use (Supplementary Equation 6), written for $[M_{avg}]$, to solve for $[M_{avg}]$ and we substitute it in (Supplementary Equation 16). Then, we solve (Supplementary Equation 16) for $[L_{free}]$ and we use this value to calculate [M] in each vesicle from (Supplementary Equation 6). With [M] and $[L_{free}]$ known, we calculate all other relevant concentrations using equations (Supplementary Equation 1) to (Supplementary Equation 4), for each vesicle.

The calculated concentrations, for every vesicle in the case of WT EGFR in the presence of EGF are plotted in Supplementary Figure 6 as a function of the total ligand concentration and the total receptor concentration in the vesicles. At low ligand concentrations unliganded monomers and dimers dominate, while at high ligand concentrations mostly single-liganded monomers and

double-liganded dimers are present. The presence of single-liganded dimers, DL, can only be observed at ligand concentrations around 10 nM EGF. The concentrations of unliganded dimers, [D], decreases with ligand concentration, as expected. Dividing each value of 2[D] by the total receptor concentration in the vesicle, [Rt], gives us the fraction of receptors in the unliganded dimeric state, f_D, in the vesicle:

$$f_D = \frac{2[D]}{[Rt]} \qquad \text{(Supplementary Equation 17)}$$

When no ligand is added to the vesicles, the observed phosphorylation, pY1068 and pY1173, is exclusively due to unliganded dimers. To quantify EGFR activation in direct response to ligand binding, $R_{phospho}$, we fit the data in Figure 2A and 3A in the main text to a Hill equation with n=1:

$$pY = R_{phosho} + k * f_D = \frac{[L_{tot}] * E_{top}}{EC_{50} + [L_{tot}]} + k * f_D \qquad \text{(Supplementary Equation 18)}$$

where f_D is given by (Supplementary Equation 17) and

$$R_{phosho} = \frac{[L_{tot}] * E_{top}}{EC_{50} + [L_{tot}]} \qquad \text{(Supplementary Equation 19)}$$

Here, pY is the measured phosphorylation, given by the fluorescence intensity of the anti-pY antibody signal divided by the EGFR-mTurq fluorescence, as shown in Figures 2A and 3A. *k* is the best-fit correction factor, which is determined in the fit, along with EC_{50} and E_{top}. Note that equation (1) for bias coefficients is valid only in the case when Hill coefficient = 1 (35), and thus we fix it to 1 in the fit.

The corrected dose response curves, $R_{phospho}$, are shown in Supplementary Figure 7. The averaged dose response curves and the fits to all the single vesicle data are shown in Supplementary Figure 8 and Supplementary Figure 9. The best-fit EC_{50} and E_{top} values are shown in Figures 2C and 3C.

The unliganded dimeric fraction, $f_{D,}$ in (Supplementary Equation 18), depends on the ligand binding constants L_1, L_2, and L_3 (Supplementary Figure 5). These constants have been measured for EGF (32). Here we use the same values for EGF and TGFα. Epiregulin is known as a low-affinity ligand, reported to bind 10 to 100 times weaker than EGF. By ITC, it has been shown to bind 46 times weaker (21). We use 46 times lower values for all three binding coefficients, L1, L2, and L3 for epiregulin-EGFR binding (21). For mouse EGF binding to EGFR, we use 3 times lower values for the three binding coefficients, L1, L2, and L3 (34).

Supplementary References

1. Chen, L., J. Placone, L. Novicky, and K. Hristova. 2010. The extracellular domain of fibroblast growth factor receptor 3 inhibits ligand-independent dimerization. Science Signaling 3:ra86.
2. Sarabipour, S., R. B. Chan, B. Zhou, G. Di Paolo, and K. Hristova. 2015. Analytical characterization of plasma membrane-derived vesicles produced via osmotic and chemical vesiculation. Biochimica et Biophysica Acta 1848:1591-1598.
3. Chen, L. R., L. Novicky, M. Merzlyakov, T. Hristov, and K. Hristova. 2010. Measuring the Energetics of Membrane Protein Dimerization in Mammalian Membranes. Journal of the American Chemical Society 132:3628-3635.
4. Del Piccolo, N., J. Placone, and K. Hristova. 2015. Effect of Thanatophoric Dysplasia Type I Mutations on FGFR3 Dimerization. Biophysical Journal 108:272-278.
5. Otsu, N. 1979. Threshold Selection Method from Gray-Level Histograms. Ieee T Syst Man Cyb 9(1):62-66.
6. Jarpe, M. B., C. Knall, F. M. Mitchell, A. M. Buhl, E. Duzic, and G. L. Johnson. 1998. [D-Arg1, D-Phe5, D-Trp7, 9, Leu11] Substance P acts as a biased agonist toward neuropeptide and chemokine receptors. Journal of Biological Chemistry 273:3097-3104.
7. Michel, M. C., and S. J. Charlton. 2018. Biased agonism in drug discovery—is it too soon to choose a path? Mol Pharmacol 93:259-265.
8. Kenakin, T. 2016. Measurement of Receptor Signaling Bias. Curr Protoc Pharmacol 74:2 15 11-12 15 15.
9. Kenakin, T. 2010. G protein coupled receptors as allosteric proteins and the role of allosteric modulators. J Recept Signal Transduct Res 30:313-321.
10. Kenakin, T., and A. Christopoulos. 2013. Signalling bias in new drug discovery: detection, quantification and therapeutic impact. Nat Rev Drug Discov 12:205-216.
11. Trevor, A. J. 2015. Chapter 2. Pharmacodynamics. In Katzung & Trevor's Pharmacology: Examination & Board Review, 11e. McGraw-Hill.
12. Berg, K. A., and W. P. Clarke. 2018. Making sense of pharmacology: inverse agonism and functional selectivity. International Journal of Neuropsychopharmacology 21:962-977.
13. Sarabipour, S., and K. Hristova. 2016. Mechanism of FGF receptor dimerization and activation. Nat Commun 7:10262.
14. Huang, Z., Y. Tan, J. Gu, Y. Liu, L. Song, J. Niu, L. Zhao, L. Srinivasan, Q. Lin, J. Deng, Y. Li, D. J. Conklin, T. A. Neubert, L. Cai, X. Li, and M. Mohammadi. 2017. Uncoupling the Mitogenic and Metabolic Functions of FGF1 by Tuning FGF1-FGF Receptor Dimer Stability. Cell Rep 20:1717-1728.
15. Belliveau, D. J., I. Krivko, J. Kohn, C. Lachance, C. Pozniak, D. Rusakov, D. Kaplan, and F. D. Miller. 1997. NGF and neurotrophin-3 both activate TrkA on sympathetic neurons but differentially regulate survival and neuritogenesis. J Cell Biol 136:375-388.
16. Kuruvilla, R., L. S. Zweifel, N. O. Glebova, B. E. Lonze, G. Valdez, H. Ye, and D. D. Ginty. 2004. A neurotrophin signaling cascade coordinates sympathetic neuron development through differential control of TrkA trafficking and retrograde signaling. Cell 118:243-255.
17. Zaccaro, M. C., H. B. Lee, M. Pattarawarapan, Z. Xia, A. Caron, P.-J. L'Heureux, Y. Bengio, K. Burgess, and H. U. Saragovi. 2005. Selective small molecule peptidomimetic

ligands of TrkC and TrkA receptors afford discrete or complete neurotrophic activities. Chem Biol 12:1015-1028.
18. Chen, D., F. Brahimi, Y. Angell, Y.-C. Li, J. Moscowicz, H. U. Saragovi, and K. Burgess. 2009. Bivalent peptidomimetic ligands of TrkC are biased agonists and selectively induce neuritogenesis or potentiate neurotrophin-3 trophic signals. ACS Chem Biol 4:769-781.
19. Harrington, A. W., C. St Hillaire, L. S. Zweifel, N. O. Glebova, P. Philippidou, S. Halegoua, and D. D. Ginty. 2011. Recruitment of actin modifiers to TrkA endosomes governs retrograde NGF signaling and survival. Cell 146:421-434.
20. Scarpi, D., D. Cirelli, C. Matrone, G. Castronovo, P. Rosini, E. G. Occhiato, F. Romano, L. Bartali, A. M. Clemente, G. Bottegoni, A. Cavalli, G. De Chiara, P. Bonini, P. Calissano, A. T. Palamara, E. Garaci, M. G. Torcia, A. Guarna, and F. Cozzolino. 2012. Low molecular weight, non-peptidic agonists of TrkA receptor with NGF-mimetic activity. Cell Death Dis 3:e339.
21. Freed, D. M., N. J. Bessman, A. Kiyatkin, E. Salazar-Cavazos, P. O. Byrne, J. O. Moore, C. C. Valley, K. M. Ferguson, D. J. Leahy, D. S. Lidke, and M. A. Lemmon. 2017. EGFR Ligands Differentially Stabilize Receptor Dimers to Specify Signaling Kinetics. Cell 171:683-695 e618.
22. Jorgensen, C., A. Sherman, G. I. Chen, A. Pasculescu, A. Poliakov, M. Hsiung, B. Larsen, D. G. Wilkinson, R. Linding, and T. Pawson. 2009. Cell-specific information processing in segregating populations of Eph receptor ephrin-expressing cells. Science 326:1502-1509.
23. Verheyen, T., T. Fang, D. Lindenhofer, Y. Wang, K. Akopyan, A. Lindqvist, B. Högberg, and A. I. Teixeira. 2020. Spatial organization-dependent EphA2 transcriptional responses revealed by ligand nanocalipers. Nucleic Acids Res 48:5777-5787.
24. Ho, C. C. M., A. Chhabra, P. Starkl, P. J. Schnorr, S. Wilmes, I. Moraga, H. S. Kwon, N. Gaudenzio, R. Sibilano, T. S. Wehrman, M. Gakovic, J. T. Sockolosky, M. R. Tiffany, A. M. Ring, J. Piehler, I. L. Weissman, S. J. Galli, J. A. Shizuru, and K. C. Garcia. 2017. Decoupling the Functional Pleiotropy of Stem Cell Factor by Tuning c-Kit Signaling. Cell 168:1041-1052 e1018.
25. Salazar-Cavazos, E., C. F. Nitta, E. D. Mitra, B. S. Wilson, K. A. Lidke, W. S. Hlavacek, and D. S. Lidke. 2020. Multisite EGFR phosphorylation is regulated by adaptor protein abundances and dimer lifetimes. Mol Biol Cell 31:695-708.
26. Kolb, P., T. Kenakin, S. P. H. Alexander, M. Bermudez, L. M. Bohn, C. S. Breinholt, M. Bouvier, S. J. Hill, E. Kostenis, K. A. Martemyanov, R. R. Neubig, H. O. Onaran, S. Rajagopal, B. L. Roth, J. Selent, A. K. Shukla, M. E. Sommer, and D. E. Gloriam. 2022. Community guidelines for GPCR ligand bias: IUPHAR review 32. Br J Pharmacol 179:3651-3674.
27. Weiss, A., and J. Schlessinger. 1998. Switching signals on or off by receptor dimerization. Cell 94:277-280.
28. Byrne, P. O., K. Hristova, and D. J. Leahy. 2020. EGFR forms ligand-independent oligomers that are distinct from the active state. The Journal of biological chemistry.
29. Needham, S. R., S. K. Roberts, A. Arkhipov, V. P. Mysore, C. J. Tynan, L. C. Zanetti-Domingues, E. T. Kim, V. Losasso, D. Korovesis, M. Hirsch, D. J. Rolfe, D. T. Clarke, M. D. Winn, A. Lajevardipour, A. H. Clayton, L. J. Pike, M. Perani, P. J. Parker, Y.

Shan, D. E. Shaw, and M. L. Martin-Fernandez. 2016. EGFR oligomerization organizes kinase-active dimers into competent signalling platforms. Nat Commun 7:13307.
30. Huang, Y., S. Bharill, D. Karandur, S. M. Peterson, M. Marita, X. Shi, M. J. Kaliszewski, A. W. Smith, E. Y. Isacoff, and J. Kuriyan. 2016. Molecular basis for multimerization in the activation of the epidermal growth factor receptor. Elife 5.
31. Chung, I., R. Akita, R. Vandlen, D. Toomre, J. Schlessinger, and I. Mellman. 2010. Spatial control of EGF receptor activation by reversible dimerization on living cells. Nature 464:783-U163.
32. Macdonald, J. L., and L. J. Pike. 2008. Heterogeneity in EGF-binding affinities arises from negative cooperativity in an aggregating system. Proceedings of the National Academy of Sciences of the United States of America 105:112-117.
33. Sanders, J. M., M. E. Wampole, M. L. Thakur, and E. Wickstrom. 2013. Molecular determinants of epidermal growth factor binding: a molecular dynamics study. PLoS One 8:e54136.
34. Nexo, E., and H. F. Hansen. 1985. Binding of epidermal growth factor from man, rat and mouse to the human epidermal growth factor receptor. Biochim Biophys Acta 843:101-106.
35. Rajagopal, S., S. Ahn, D. H. Rominger, W. Gowen-MacDonald, C. M. Lam, S. M. Dewire, J. D. Violin, and R. J. Lefkowitz. 2011. Quantifying ligand bias at seven-transmembrane receptors. Mol Pharmacol 80:367-377.
