# Supplementary material for: Quantification of ligand and mutation-induced bias in EGFR phosphorylation in direct response to ligand binding
Source: Nat Commun. 2023 Nov 21;14:7579. doi: 10.1038/s41467-023-42926-8 (PMC10663608; doi:10.1038/s41467-023-42926-8)
Supplement: Supplementary file 3 — Reporting Summary [file 41467_2023_42926_MOESM3_ESM.pdf]

nature portfolio

Corresponding author(s): Kalina Hristova

Last updated by author(s): Oct 16, 2023

Reporting Summary

Nature Portfolio wishes to improve the reproducibility of the work that we publish. This form provides structure for consistency and transparency in reporting. For further information on Nature Portfolio policies, see our Editorial Policies and the Editorial Policy Checklist.

Statistics

For all statistical analyses, confirm that the following items are present in the figure legend, table legend, main text, or Methods section.

n/a	Confirmed	
☐	☒	The exact sample size (*n*) for each experimental group/condition, given as a discrete number and unit of measurement
☐	☒	A statement on whether measurements were taken from distinct samples or whether the same sample was measured repeatedly
☐	☒	The statistical test(s) used AND whether they are one- or two-sided<br>*Only common tests should be described solely by name; describe more complex techniques in the Methods section.*
☒	☐	A description of all covariates tested
☒	☐	A description of any assumptions or corrections, such as tests of normality and adjustment for multiple comparisons
☐	☒	A full description of the statistical parameters including central tendency (e.g. means) or other basic estimates (e.g. regression coefficient) AND variation (e.g. standard deviation) or associated estimates of uncertainty (e.g. confidence intervals)
☐	☒	For null hypothesis testing, the test statistic (e.g. *F*, *t*, *r*) with confidence intervals, effect sizes, degrees of freedom and *P* value noted<br>*Give P values as exact values whenever suitable.*
☒	☐	For Bayesian analysis, information on the choice of priors and Markov chain Monte Carlo settings
☒	☐	For hierarchical and complex designs, identification of the appropriate level for tests and full reporting of outcomes
☒	☐	Estimates of effect sizes (e.g. Cohen's *d*, Pearson's *r*), indicating how they were calculated

Our web collection on statistics for biologists contains articles on many of the points above.

Software and code

Policy information about availability of computer code

Data collection	Leica LASX for image acquisition version 3.5.6.21594, Nanodrop 2000c 1.5, Biotek Gen5 Version 2.05
Data analysis	custom written MATLAB 2020a code, Graphpad Prism9, https://figshare.com/articles/dataset/Quantification_of_ligand_and_mutation-induced_bias_in_EGFR_phosphorylation_in_direct_response_to_ligand_binding/24162846

For manuscripts utilizing custom algorithms or software that are central to the research but not yet described in published literature, software must be made available to editors and reviewers. We strongly encourage code deposition in a community repository (e.g. GitHub). See the Nature Portfolio guidelines for submitting code & software for further information.

Data

Policy information about availability of data

All manuscripts must include a data availability statement. This statement should provide the following information, where applicable:

- Accession codes, unique identifiers, or web links for publicly available datasets
- A description of any restrictions on data availability
- For clinical datasets or third party data, please ensure that the statement adheres to our policy

The data can be found here: https://figshare.com/articles/dataset/Quantification_of_ligand_and_mutation-induced_bias_in_EGFR_phosphorylation_in_direct_response_to_ligand_binding/24162846
There are no restrictions on data availability

Research involving human participants, their data, or biological material

Policy information about studies with human participants or human data. See also policy information about sex, gender (identity/presentation), and sexual orientation and race, ethnicity and racism.

Reporting on sex and gender	n/a
Reporting on race, ethnicity, or other socially relevant groupings	n/a
Population characteristics	n/a
Recruitment	n/a
Ethics oversight	n/a

Note that full information on the approval of the study protocol must also be provided in the manuscript.

Field-specific reporting

Please select the one below that is the best fit for your research. If you are not sure, read the appropriate sections before making your selection.

☒ Life sciences ☐ Behavioural & social sciences ☐ Ecological, evolutionary & environmental sciences

For a reference copy of the document with all sections, see nature.com/documents/nr-reporting-summary-flat.pdf

Life sciences study design

All studies must disclose on these points even when the disclosure is negative.

Sample size	Dose response data are from 11570 single vesicles over 25 independent experiments for WT and 8,009 vesicles for L834R EGFR in 23 independent experiments. Transducer function measurements are from 3,085 individual vesicles over 9 independent experiments. Since errors are due to white noise, we collect data until the Gaussian distribution of data for each ligand concentration is sufficiently sampled.
Data exclusions	Occasional bad pixels in thousands of vesicle images would give unreasonable high signals. Any outliers in the datasets were removed with Matlab`s rmoutliers function in the standard settings.
Replication	At least 3 independent experiments were always performed. All experimental findings were reproducible.
Randomization	Each experimental group was comprised by vesicles imaged in the presence of the same ligand concentrations. The order of the imaging of the different groups was varied to exclude any time effects. All imaging was performed after one hour of ligand addition in agreement with the kinetics experiment. The analysis was automated in order to eliminate bias by the researcher.
Blinding	No blinding was carried out for data collection and analysis. All experiments were assigned into groups, including control groups, and all data was analyzed automatically. Thus, analysis was done objectively and without bias.

Reporting for specific materials, systems and methods

We require information from authors about some types of materials, experimental systems and methods used in many studies. Here, indicate whether each material, system or method listed is relevant to your study. If you are not sure if a list item applies to your research, read the appropriate section before selecting a response.

Materials & experimental systems

n/a	Involved in the study	
☐	☒	Antibodies
☐	☒	Eukaryotic cell lines
☒	☐	Palaeontology and archaeology
☒	☐	Animals and other organisms
☒	☐	Clinical data
☒	☐	Dual use research of concern
☒	☐	Plants

Methods

n/a	Involved in the study	
☒	☐	ChIP-seq
☒	☐	Flow cytometry
☒	☐	MRI-based neuroimaging

Antibodies

Antibodies used	Alexa488-labeled anti-pY1068 EGFR antibody (IC3570G100, R&D Systems)<br>Alexa488-labeled anti-pY1173 EGFR antibody (NBP1-44893AF488, Novus Biologicals)<br>FITC-labeled anti pY 4G10 antibody (05-321, MilliPore Sigma)
Validation	These antibodies have been used successfully in the literature:<br>FITC-labeled anti-pY 4G10 antibody (05-321, Sigma Aldrich) [Stone, M. B., and S. L. Veatch. 2015. Steady-state cross-correlations for live two-colour super-resolution localization data sets. Nat Commun 6:7347.]<br><br>Anti p-Y1068 EGFR antibody (IC3570G100, R&D Systems) [Vasudevan, S., I. A. Adejumobi, H. Alkhatib, S. Roy Chowdhury, S. Stefansky, A. M. Rubinstein, and N. Kravchenko-Balasha. 2021. Drug-Induced Resistance and Phenotypic Switch in Triple-Negative Breast Cancer Can Be Controlled via Resolution and Targeting of Individualized Signaling Signatures. Cancers (Basel) 13.]<br><br>Alexa488-labeled anti phospho-Y1173 EGFR antibody (NBP1-44893AF488, Novus Biologicals) [Yao, T. W., J. Zhang, M. Prados, W. A. Weiss, C. D. James, and T. Nicolaides. 2017. Acquired resistance to BRAF inhibition in BRAFV600E mutant gliomas. Oncotarget 8:583-595.]

Eukaryotic cell lines

Policy information about cell lines and Sex and Gender in Research

Cell line source(s)	CHO cells from ATCC
Authentication	not authenticated
Mycoplasma contamination	not tested
Commonly misidentified lines (See ICLAC register)	No commonly misidentified lines were used

Plants

Seed stocks	n/a
Novel plant genotypes	n/a
Authentication	n/a
